# Supplementary material for: Identifying the tilt angle and correcting the orbital angular momentum spectrum dispersion of misaligned light beam
Source: Sci Rep. 2017 Aug 11;7:7873. doi: 10.1038/s41598-017-07734-3 (PMC5554247; doi:10.1038/s41598-017-07734-3)
Supplement: Supplementary file 1 — Supplementary information [file 41598_2017_7734_MOESM1_ESM.pdf]

Supplementary Information

Identifying the tilt angle and correcting the orbital angular momentum spectrum dispersion of misaligned light beam

Peng Zhao, Shikang Li, Yu Wang, Xue Feng, Kaiyu Cui, Fang Liu, Wei Zhang, and Yidong Huang*

Department of Electronic Engineering, Tsinghua National Laboratory for Information Science and Technology, Tsinghua University, Beijing, China

x-feng@tsinghua.edu.cn

Detailed Deduction of the equations:

The deduction of the Eq. (11) from Eq. (10) in the main text:

$$I_{inter}(r,\phi)=I_{obj}(r,\phi)+I_{ref}(r,\phi)+2\sqrt{I_{obj}(r,\phi)I_{ref}(r,\phi)}\cos(\varphi_{obj}(r,\phi)-\varphi_{ref}(r,\phi)-\varphi_{del}) \quad \text{(S1)}$$

Putting in the φ_{del1} and φ_{de2}, with the relation of $\varphi_{del2=}\varphi_{del1}+\pi/2$, the interference patterns, denoted as I_{inter1} and I_{inter2} can be deduced as:

$$I_{inter1}(r,\phi)=I_{obj}(r,\phi)+I_{ref}(r,\phi)+2\sqrt{I_{obj}(r,\phi)I_{ref}(r,\phi)}\cos(\varphi_{obj}(r,\phi)-\varphi_{ref}(r,\phi)-\varphi_{del1}) \quad \text{(S2)}$$

$$\begin{aligned}I_{inter2}(r,\phi)&=I_{obj}(r,\phi)+I_{ref}(r,\phi)+2\sqrt{I_{obj}(r,\phi)I_{ref}(r,\phi)}\cos(\varphi_{obj}(r,\phi)-\varphi_{ref}(r,\phi)-\varphi_{del2})\\&=I_{obj}(r,\phi)+I_{ref}(r,\phi)+2\sqrt{I_{obj}(r,\phi)I_{ref}(r,\phi)}\sin(\varphi_{obj}(r,\phi)-\varphi_{ref}(r,\phi)-\varphi_{del1})\end{aligned} \quad \text{(S3)}$$

Then:

$$\begin{aligned}2\sqrt{I_{obj}(r,\phi)I_{ref}(r,\phi)}\cos(\varphi_{obj}(r,\phi)-\varphi_{ref}(r,\phi)-\varphi_{del1})&=I_{inter1}(r,\phi)-I_{obj}(r,\phi)-I_{ref}(r,\phi)\\2\sqrt{I_{obj}(r,\phi)I_{ref}(r,\phi)}\sin(\varphi_{obj}(r,\phi)-\varphi_{ref}(r,\phi)-\varphi_{del1})&=I_{inter2}(r,\phi)-I_{obj}(r,\phi)-I_{ref}(r,\phi)\end{aligned} \quad \text{(S4)}$$

Since the phase delay of φ_{del1} is constant, ignore the term without loss the generality, the phase difference between the object light beam and the reference light beam can be deduced as the Eq. (10):

$$\varphi_{obj}(r,\phi)-\varphi_{ref}(r,\phi)=\arg(I_{inter1}(r,\phi)-I_{obj}(r,\phi)-I_{ref}(r,\phi),I_{inter2}(r,\phi)-I_{obj}(r,\phi)-I_{ref}(r,\phi)) \quad \text{(S5)}$$

Measurement of the tilt angle:

Under the Cartesian coordinates (x, y, z), the light field of the Gaussian beam propagating along the z-axis can be expressed as:

$$E(x,y,z)=E_0\frac{\omega_0}{\omega(z)}\exp\left(\frac{-(x^2+y^2)}{\omega(z)^2}\right)\exp\left(ik\frac{(x^2+y^2)}{2R(z)}\right)\exp(ikz), \quad \text{(S6)}$$

where E_0 denotes normalized coefficient of the field, while ω_0 and $\omega(z)$ denotes the size of the light waist and light spot at the position of z, respectively. $R(z)$ and k denotes the curvature radius of the wavefront at z and the wave vector.

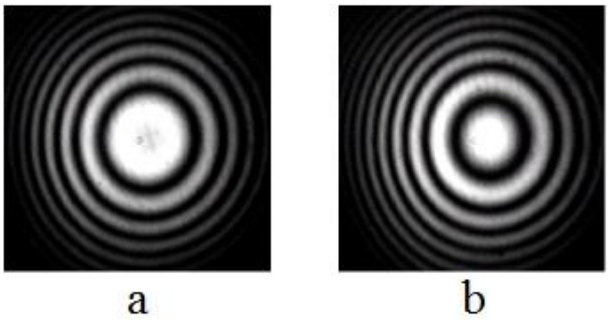

Fig. S1. The interference patterns of two Gaussian beams, while there is without tilt and with tilt between the two Gaussian beams in (a) and (b).

Considering two Gaussian beams with the likely intensity distribution but different phase distribution, from different $R(z)$. The dark fringe positions in the interference pattern of such two Gaussian beams is mainly the positions of the phase difference between two beams is odd times of π. Thus the position function of the dark fringes can be expressed as:

$$\frac{1}{2}k\left(\frac{x^2+y^2}{R_1(z)}-\frac{x^2+y^2}{R_2(z)}\right)=(2n+1)\pi, \tag{S7}$$

where $R_1(z)$ and $R_2(z)$ denotes the curvature radiuses of the wavefront at z of the two Gaussian beams. Determined by the equation, the dark fringes are several concentric rings with the center at position $(x, y)=(0, 0)$, as shown in Fig. S1(a). The coefficient of $\frac{1}{R_1(z)}-\frac{1}{R_2(z)}$ determines the intervals between the rings. Thus the coefficient could be extracted from the distribution the rings. In our experiments, the two Gaussian beams come from objective light and reference light, respectively. Considering the objective light having a tilt between reference light as the situation shown in the Fig. 2 in the main body, the phase distribution of the objective light would add an extra phase $\Delta\varphi\ (x, y)=k\theta x$, according to the analysis in the last part of the introduction section. The dark fringes position function of the interference pattern could be deduced as:

$$\frac{1}{2}k\left(\frac{x^2+y^2}{R_1(z)}+\theta x-\frac{x^2+y^2}{R_2(z)}\right)=(2n+1)\pi. \tag{S8}$$

Thus, the center of the concentric rings shifts from 0 to $-\frac{\theta}{2\left(\frac{1}{R_1(z)}-\frac{1}{R_2(z)}\right)}$, while the intervals between the rings remains, as shown in Fig. S1(b). The tilt angle of the objective light beam can be extracted from such position shift.

To demonstrate the generality of our method, pure OAM state beams of -3~3-order and several superposition OAM beams are adopted as the objective light beam, respectively. Here we show the results of OAM spectrum correction.

Beams carrying OAM of pure state:

Figure S2 shows the measured OAM spectrums and the corrected OAM spectrums. (a), (c), (e), (g), (i), (k) and (m) shows the measured OAM spectrums of beams carrying OAM of l=-3~3, respectively, while (b), (d), (f), (h), (j), (l) and (n) shows the corresponding corrected OAM spectrums. In figures of measured OAM spectrums, the tilt angle θ and dispersion factor υ are labeled while in figures of corrected OAM spectrums, only the dispersion factor υ are labeled.

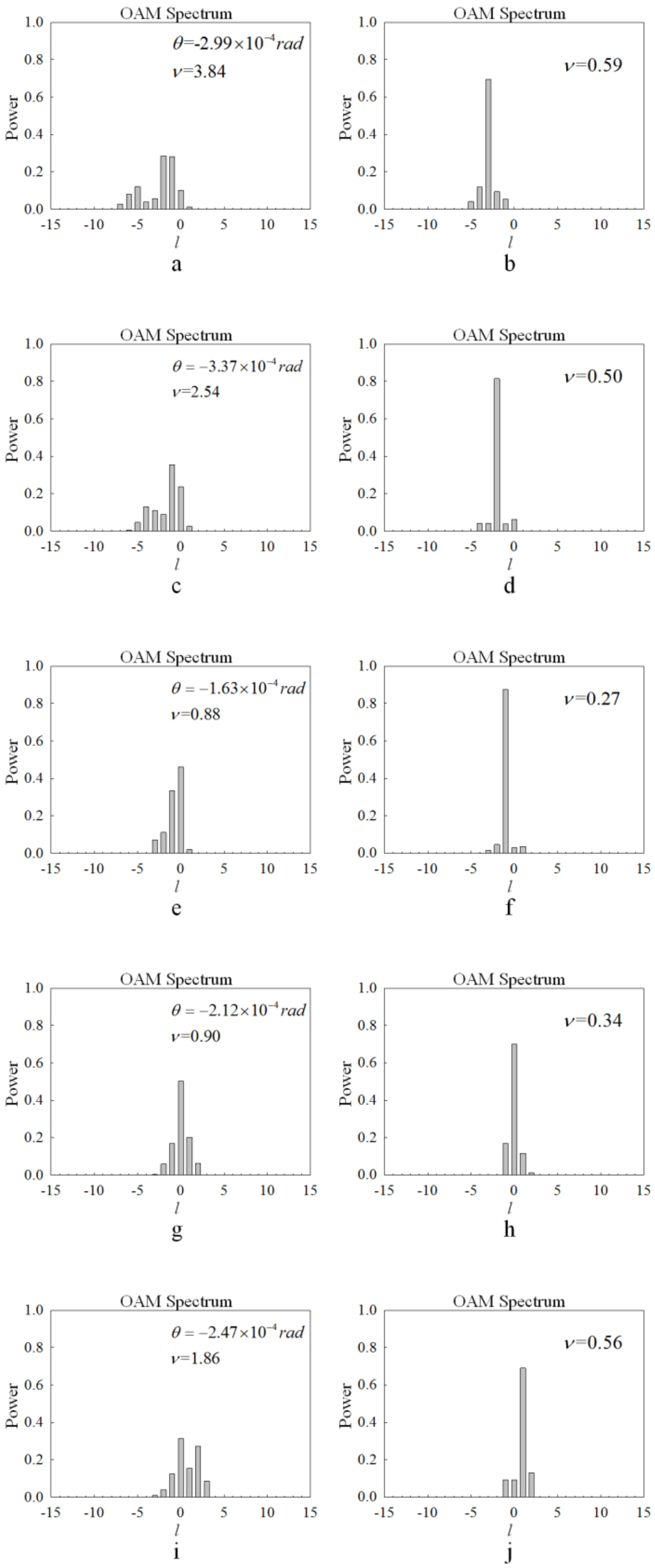

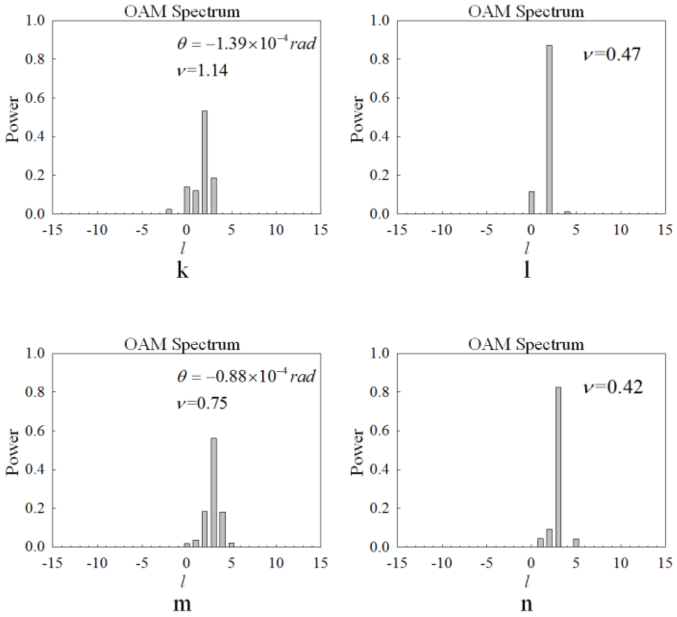

Fig. S2. The measured OAM spectrums and the corrected OAM spectrums. (a), (c), (e), (g), (i), (k) and (m) shows the measured OAM spectrums of beams carrying OAM of *l*=-3~3, respectively, while (b), (d), (f), (h), (j), (l) and (n) shows the corresponding corrected OAM spectrums.

Beams carrying OAM of Superposition states:

Figure S3(a) shows the measured OAM spectrum of beam superposed by OAM of *l*=3 and 0 with tilt angle of -4.60×10^{-4}*rad*, while (c) shows the measured OAM spectrum of beam superposed by OAM of *l*=-3 and 3 with tilt angle of -4.15×10^{-4}*rad*. Figure S3(b) and (d) show the corrected OAM spectrum corresponding to (a) and (c), respectively.

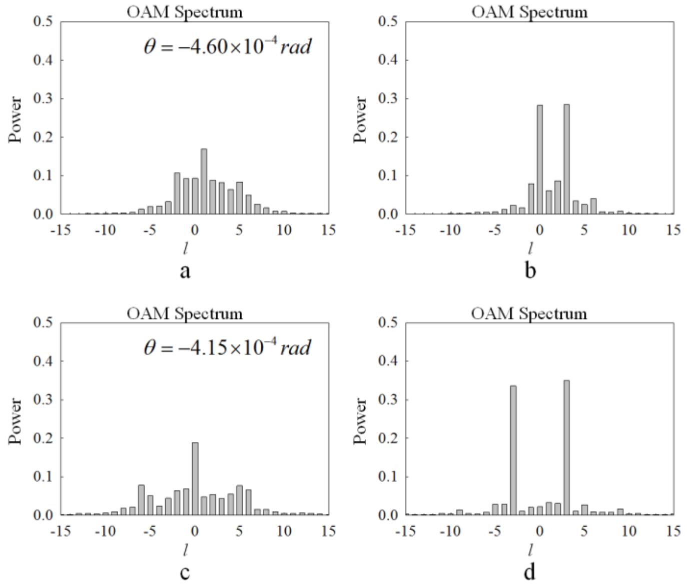

Fig. S3. (a) shows the measured OAM spectrum of beam superposed by OAM of *l*=3 and 0 with tilt angle of -4.60×10^{-4}*rad*, while (c) shows the measured OAM spectrum of beam superposed by OAM of *l*=-3 and 3 with tilt angle of -4.15×10^{-4}*rad*. (b) and (d) show the corrected OAM spectrum corresponding to (a) and (c), respectively.
